# Targeted Orexin and Hypothalamic Neuropeptides for Migraine

**DOI:** 10.1007/s13311-017-0602-3

**Published:** 2018-02-13

**Authors:** Lauren C. Strother, Anan Srikiatkhachorn, Weera Supronsinchai

**Affiliations:** 1 Headache Group, Basic and Clinical Neuroscience, Institute of Psychiatry, Psychology and Neuroscience, King’s College London, London, UK; 2 International Medical College, King Mongkut’s Institute of Technology Ladkrabang, Bangkok, Thailand; 3 Department of Physiology, Faculty of Dentistry, Chulalongkorn University, Pathumwan, Bangkok Thailand

**Keywords:** Hypothalamus, migraine, orexin, oxytocin, neuropeptide Y (NPY), pituitary adenylate cyclase activating protein (PACAP)

## Abstract

The hypothalamus is involved in the regulation of homeostatic mechanisms and migraine-related trigeminal nociception and as such has been hypothesized to play a central role in the migraine syndrome from the earliest stages of the attack. The hypothalamus hosts many key neuropeptide systems that have been postulated to play a role in this pathophysiology. Such neuropeptides include but are not exclusive too orexins, oxytocin, neuropeptide Y, and pituitary adenylate cyclase activating protein, which will be the focus of this review. Each of these peptides has its own unique physiological role and as such many preclinical studies have been conducted targeting these peptide systems with evidence supporting their role in migraine pathophysiology. Preclinical studies have also begun to explore potential therapeutic compounds targeting these systems with some success in all cases. Clinical efficacy of dual orexin receptor antagonists and intranasal oxytocin have been tested; however, both have yet to demonstrate clinical effect. Despite this, there were limitations in these cases and strong arguments can be made for the further development of intranasal oxytocin for migraine prophylaxis. Regarding neuropeptide Y, work has yet to begun in a clinical setting, and clinical trials for pituitary adenylate cyclase activating protein are just beginning to be established with much optimism. Regardless, it is becoming increasingly clear the prominent role that the hypothalamus and its peptide systems have in migraine pathophysiology. Much work is required to better understand this system and the early stages of the attack to develop more targeted and effective therapies aimed at reducing attack susceptibility with the potential to prevent the attack all together.

**Electronic supplementary material:**

The online version of this article (10.1007/s13311-017-0602-3) contains supplementary material, which is available to authorized users.

## Introduction

The hypothalamus is involved in the modulation of a number of fundamental physiological processes, including sleep, circadian rhythms, appetite, thirst, urination, and the regulation of the autonomic, cardiovascular, endocrine, and trigeminal pain systems [1]. Modulation of such a variety of physiological processes is achieved through multiple peptide systems working synergistically and disruption of these systems can result in physiological dysfunction. Migraine is largely considered a disorder of disrupted homeostasis where disturbances in circadian rhythms, sleep, feeding (and thus peptide systems that regulate these systems), can both trigger and be symptomatic of the attack. As many of these functions are under hypothalamic control, aberrant hypothalamic mechanisms likely play a role in migraine pathogenesis from the early stages and throughout.

The opportunity to identify and target the early stages of the attack is an exciting step forward in migraine management and treatment. Currently, the majority of therapies are ineffective or poorly tolerated and largely focus on treating the head pain component of the disorder [2]. By shifting our focus to the early mechanisms, there is potential to identify novel treatment targets aimed at reducing attack susceptibility and ultimately preventing the attack all together.

## Hypothalamic Involvement in Migraine

As alluded to in the introduction, the hypothalamic involvement in migraine is complex with dysfunction evident from the earliest stages and throughout the different phases of the migraine attack. The first section of this review will thus highlight the general hypothalamic involvement throughout each phase before exploring the unique roles of its peptide systems and the current targeted pharmacotherapies.

### Premonitory and Autonomic Symptoms

Premonitory symptoms occur up to 72 h before the onset of migraine pain [3]. Such symptoms are likely hypothalamic in origin and include changes in sleep, arousal, mood, appetite, urination, and yawning [4, 5]. Maniyar et al. [6] first described abnormal activation of the hypothalamus during the premonitory phase, before the onset of head pain. Here, in a model of experimental migraine, migraine patients were observed to have an increase in hypothalamic blood flow during the presence of premonitory symptoms and in the absence of head pain [6]. This aberrant hypothalamic activity in preictal phase was corroborated in spontaneous attacks. Here, Schulte et al. [7] identified increased hypothalamic activation, in addition to increased activation of the spinal trigeminal nucleus and middle pons, in the 24 h before the onset of migraine head pain [7], thus confirming a functional role for the hypothalamus in generating migraine attacks*.*

As the attack progresses, autonomic symptoms, including nausea, nasal congestion, and lacrimation, may develop in addition to head pain. Aberrant hypothalamic activity, particularly a hypersensitivity to dopamine, may largely account for symptoms of nausea, vomiting, and excessive yawning. This has been supported experimentally, where administration of dopamine agonists increase these symptoms in migraine patients [8]. Furthermore, autonomic symptoms may also arise through hypothalamic descending projections activating the superior salivatory nucleus, which is thought to regulate autonomic function [9–11].

### Migraine Nociception

Migraine pain is centered around aberrant activation of the trigeminovascular system leading to an overall dysregulation of sensory processing. Pseudo-unipolar trigeminal afferents innervate pain-sensing extracranial structures while sending central projections to the trigeminal cervical complex (TCC) of the brainstem [12, 13]. Second-order neurons from the TCC send ascending afferents to the thalamus where periphery sensory and nociceptive information is integrated and processed before being relayed to somatosensory, visual, auditory, and motor cortical areas via third-order thalamocortical neurons. The TCC also sends additional projections directly to other pain-modulating structures, such as the hypothalamus, locus coeruleus (LC), and periaqueductal grey (PAG) [12, 14, 15]. Additionally, the TCC is under descending control from direct cortico-hypothalamic projections [15, 16] or indirectly through PAG–rostroventromedial medulla projections that can further act to facilitate or inhibit TCC activity and thus nociceptive signaling [3, 17].

The hypothalamus is known to be involved in nociceptive processing and is linked with key areas of the pain neuro-axis, including the cortex, thalamus, amygdala, PAG, and the spinal cord dorsal horn [18]. As such, stimulation of several hypothalamic nuclei such as the lateral, anterior, and paraventricular nucleus of the hypothalamus have been shown to be antinociceptive [19–22].

In specific regard to migraine-related pain, imaging studies have shown hypothalamic activation during the pain phase of the migraine attack [23]. The hypothalamus is thought to modulate trigeminal pain through reciprocal connections with the TCC and further direct and indirect descending pathways through other pain regulatory structures such as the PAG and the LC [15, 24, 25]. This is supported experimentally, where modulation of the hypothalamus has been shown to regulate trigeminal nociceptive processing [15, 24, 25] and reciprocally, stimulation of pain sensing extracranial structures produces c-Fos, a neuronal marker of activation, in hypothalamic nuclei [14, 26]. Furthermore, it has been shown that in spontaneous attacks, functional connectivity switches from the TCC to PAG synchrony in the 24 h leading up to the head pain [27], suggesting that this change could possibly drive the transition between migraine phases.

### Increased Migraine Susceptibility

Hypothalamic-related perturbations, such as sleep disturbances, changes in arousal, and appetite dysregulation, can increase the probability of an attack in addition to being an indication of early symptoms. Sleep and feeding behaviors are regulated by both circadian periodicity and individual mechanisms of which dysfunction could contribute to migraine pathophysiology.

Increasing evidence from clinical and basic scientific research suggests that circadian rhythm dysregulation plays a significant role in migraine susceptibility. The hypothalamus, specifically the suprachiasmatic nucleus, controls the circadian periodicity of biological processes such as sleep–wake, appetite, metabolism, and thermoregulation [28]. Alterations in circadian cycle may alter the threshold for attack initiation, which is supported by hypothalamic modulation of nociceptive processing [24, 25]. Furthermore, migraine shows a pronounced circadian pattern of attack onset with a population preponderance in the morning [29, 30], especially in chronic migraine, whereas patients exhibit abnormal circadian levels of cortisol [31].

In clinical practice, patients with migraine commonly report sleep disturbances and circadian disruption as reliable triggers. This can include too much or too little sleep, jetlag, and shift work [32, 33]. Patients also complain about abnormal sleeping patterns [34] but praise sleep itself as an abortive strategy [35, 36]. Additionally, migraine and other headache disorders are associated with sleep disorders, such as insomnia [37] and narcolepsy [38], and circadian sleep disorders of advanced [39] and delayed [40] sleep phase.

Feeding and appetite also exhibit circadian rhythmicity controlled by the hypothalamus and have been implicated in increased migraine susceptibility and attack symptomology. For example, skipping meals is a common migraine trigger, and attack frequency and severity have been seen to increase with body mass index [5, 41–43]. Furthermore, migraineurs show altered fasting glucose and insulin levels [44–46] and, anatomically, appetite regulating hypothalamic nuclei make connections with the TCC and are thought to contribute to migraine symptoms such as loss of appetite in addition to pain modulation [47].

## Hypothalamic Neuropeptides in Migraine

As we have seen in the previous section, the hypothalamus is deeply involved in the migraine attack, including increasing susceptibility, attack triggering, and symptomology. What we have not explored yet is how the hypothalamus is able to control and regulate such a wide variety of physiological functions. The hypothalamus is an extremely heterogeneous nucleus that synthesizes many neuropeptides. Each uniquely contribute to the regulation of physiological functions such as sleep–wake regulation, appetite, and nociceptive processing, and thus individual peptide systems have been proposed to play a key role in the pathogenesis of migraine. This section will explore the role of orexins, pituitary adenylate cyclase activating protein (PACAP), oxytocin, and neuropeptide Y (NPY), and highlight preclinical and clinical therapies specifically targeting these systems.

### Orexins

#### Orexin Physiology

The orexins (alternatively called hypocretins) are a pair of hypothalamic neuropeptides synthesized in cell bodies exclusively in the lateral hypothalamus [48, 49]. The 2 peptides, orexin A (OXA) and orexin B (OXB), are cleaved from the same precursor, prepro-orexin, and undergo post-translational modifications to result in a 33- and a 28-residue peptide, respectively. Both peptides have an excitatory effect on 2 G protein-coupled receptors (GPCRs), orexin receptor 1 (OXR1) and orexin receptor 2 (OXR2). While OXA shows equal affinity for both receptors, OXB shows a 10-fold preferential affinity for OXR2 [49]. Orexinergic neurons project to a wide variety of brain areas with receptor expression largely in agreement with fiber projections [50]; however, some brain areas can exclusively express either OXR1 such as the LC, or OXR2 such as tuberomammillary nucleus and rostral ventromedial medulla [51].

Such widespread projections implicate the orexins and thus the hypothalamus in the modulation of homeostatic functions, including appetite, sleep–wake, hormone secretion, and autonomic regulation [52–55]. With regard to feeding, activation of the arcuate nucleus by orexins can stimulate feeding behavior [49, 56–58], which, in turn, is modulated by peripheral feeding hormones such as leptin and glucose, thus highlighting a possible role in energy homeostasis [58, 59]. Conversely, under conditions of food deprivation, orexin receptor and protein expression increase, with prepro-orexin levels seen to rise 48 h after fasting [60, 61].

Orexin levels experience a circadian periodicity where they tend to be highest during awake periods [54, 55]. Furthermore, the role of orexin in sleep and wakefulness is thought to be centered on stabilization of the sleep–wake transition. Direct excitation of monoaminergic and cholinergic hypothalamic brainstem networks such as noradrenergic neurons in the LC actively promotes wakefulness [62–65], whereas a general loss of orexin neurotransmission results in disrupted sleep–wake regulation as seen in the sleep disorder narcolepsy [66].

#### Orexins and Migraine

The central role that orexins play in the regulation of homeostatic mechanisms points to a potential role for orexin disruption in migraine pathophysiology. Such a role has been supported by increasing clinical and anatomical evidence. Migraineurs, both chronic and episodic, have shown altered levels of orexin as measured in the cerebrospinal fluid (CSF). Episodic migraineurs have been shown to have lower levels, whereas patients with chronic and medication overuse headache exhibit higher levels [67], both of which support a dysfunction in orexin physiology.

Perhaps the most convincing clinical association is in the increased prevalence of migraine in narcoleptic patients (females 64% and males 45% *vs* 30% and 8% in the general population) [38], indicating that loss of orexinergic neurotransmission may contribute to the pathogenesis of migraine in these individuals.

Anatomically, the orexin system is closely integrated with other brain systems involved in migraine pathophysiology. Hypothalamic orexinergic afferents widely project to many brain areas involved in trigeminal nociception, including the cerebral cortex, cingulate cortex, paraventricular thalamic nuclei, LC, PAG, nucleus raphe magnum, and spinal and trigeminal dorsal horns [50, 68, 69]. Specifically, hypothalamic orexinergic afferents have been shown to project to the TCC in the brain stem both directly and indirectly through the PAG, which has previously been shown to modulate trigeminal nociceptive processing [70]. Orexins are also thought to be involved in the transition of hypothalamic functional connectivity from the TCC to the PAG 24 h prior to the onset of an attack, as previously mentioned. Here, orexins are thought to promote the switch from direct modulation of TCC to the PAG by recruiting descending antinociceptive PAG networks in an attempt to combat increased TCC activity that increase as an attack approaches [27]. Furthermore, orexinergic cells receive robust efferents from limbic structures [71], suggesting a further point of convergence for the integration of emotional stimuli on arousal and pain states relevant to migraine.

Taken together, disrupted orexinergic systems can contribute to altered homeostatic mechanisms that can influence attack susceptibility, premonitory and associated symptoms, and migraine nociception; as such, this system has been identified as a potential therapeutic target for migraine.

#### Migraine Drugs Targeting Orexins

Dual orexin receptor antagonists (DORAs) have already been developed for the treatment for insomnia [72] and given that migraine and sleep are intimately interconnected [32–34, 36], it was hypothesized that dual orexin receptor antagonism may also be therapeutic in migraine.

##### Preclinical Studies

As the name suggests, DORAs have equal affinity for both orexin receptors. DORA-12, commonly known as suvorexant, antagonizes an orexin-induced calcium increase in cells expressing OXR1 or OXR2 [73]. Preclinically, suvorexant is orally bioavailable, highly brain penetrant, and has high orexin receptor occupancy [73]. In preclinical models of migraine nociception, DORA-12 has been shown to attenuate trigeminal nociceptive activity. Cady et al. [74] showed that DORA-12 inhibited sensory neuronal activation in the trigeminal ganglion after the injection of Complete Freund’s Adjuvant to induce inflammation into the temporomandibular joint in the rat [74], and Hoffman et al. [75] saw DORA-12 to further attenuate trigeminal neuronal activation in response to electrical stimulation of dural trigeminal afferents. In a preclinical model of migraine aura measuring cortical spreading depression, DORA-12 was also successful in increasing the thresholds required to induce cortical spreading depression events [75]. Taken together, these data suggest that targeting the hypothalamic orexinergic system may offer a novel mechanism for preventative treatment of migraine with and without aura.

However, there has been some concern over the clinical efficacy of dual orexin receptor antagonism in migraine therapy. The selective targeting of individual receptors may prove to be more efficacious as OXA and OXB have consistently shown to have differential experimental effects on trigeminal nociception. Bartsch et al. [24] demonstrated that microinjection of OXA into the posterior hypothalamus inhibited dural-evoked trigeminal activation, whereas OXB facilitated the response. Systemic OXA also produced an antinociceptive effect and administration of OXA, but not OXB, resulted in the inhibition of vasodilation in a model of neurogenic dural vasodilation [76, 77]. Both mechanisms were determined to be modulated by OXR1 activation, as selective OXR1 antagonism blocked the effects. Conversely, microinjection of OXA and OXB into the nucleus raphe magnum, a structure involved in the tonic descending modulation of neuronal firing in the TCC, facilitates neuronal activity in the TCC—an effect predominately driven by OXR2 receptors [78].

##### Clinical Studies

Although several preclinical studies have demonstrated the possible beneficial therapeutic effect of orexin-related chemicals, only 1 randomized, double-blind, placebo-controlled clinical study has been conducted. Chabi et al. [79] investigated the effectiveness of filorexant (MK-6069), a dual (OXR1 and OXR2) receptor antagonist (DORA), in migraine prophylaxis. They found that, of 97 patients treated with filorexant (10 mg nightly) and 101 treated with placebo, there was no statistically significant difference between treatments for change from baseline in mean monthly migraine days [filorexant = –1.7, placebo = –1.3, difference = –0.4 (95% confidence interval –1.3 to 0.4)] or headache days [filorexant = –1.7, placebo = –1.2, difference = –0.5 (95% confidence interval –1.4 to 0.4)]. The authors concluded that the study failed to provide evidence that antagonism of orexin receptors with filorexant, when administered at night, is effective for migraine prophylaxis. However, these negative results may be caused by the nighttime dosing and short half-life (3–5 h) of filorexant [79, 80], and selective targeting of individual receptors as mentioned above may offer potential therapeutic benefit and is worth investigating further.

### Oxytocin

#### Oxytocin Physiology

Oxytocin is a hypothalamic neuropeptide and peptide hormone that plays a diverse role in physiological functions, including social bonding, sexual reproduction, childbirth, and modulation of pain processing. Oxytocin is a small polypeptide of only 9 amino acids and is synthesized in magnocellular neurosensory cells in the supraoptic and paraventricular nuclei of the hypothalamus [81, 82]. As oxytocin can have both endocrine and neuronal functions, it is released either into systemic circulation from the posterior pituitary [83] or by paraventricular neurons projecting to brain areas where it activates a class I GPCR [84].

#### Oxytocin and Migraine

Many brain areas involved in pain modulation have been shown to express oxytocin receptors such as the dorsal root and trigeminal ganglia, thus suggesting a role in migraine-specific pain modulation [85]. In clinical experience, high levels of oxytocin have been anecdotally linked to a reduction in migraine frequency. For example, female patients with migraine report a reduction in migraine attacks while pregnant [86, 87]; those who breast feed their babies demonstrate a lower rate of postpartum migraine recurrence than those who bottle feed [88, 89]; and 46% of women report that sex can provide migraine relief [90, 91]. Furthermore, in one particular case study of a woman with severe migraine, oxytocin infusion to induce uterine contraction alleviated migraine pain [92].

In support of this postulated role for oxytocin migraine nociception, trigeminal afferents have been shown to co-express oxytocin receptors and calcitonin gene-related peptide (CGRP) [85], a neuropeptide that has proven to be critical in the pathogenesis of migraine [93–95]. Furthermore, administration of oxytocin *in vitro* has been shown to block CGRP release, indicating that oxytocin agonists may be a novel therapeutic target for migraine [85].

Preclinical studies have subsequently demonstrated analgesic effects of oxytocin in pain processing with some indication of efficacy on trigeminovascular nociception. In agreement with animal models of pain having previously demonstrated oxytocin to be a strong analgesic [22, 96, 97], oxytocin attenuates firing of trigeminal nerves *in vitro* and produce analgesia *in vivo* in pre-existing states of inflammation or injury [98]. Furthermore, oxytocin, through its receptor, was seen to inhibit peripheral-evoked neural activity at the level of the TCC in rat [99]. Therefore, taken together, it is reasonable to hypothesize that targeting oxytocin may have therapeutic effect in migraine treatment.

#### Migraine Drugs Targeting Oxytocin

Recent work has begun to investigate intranasal oxytocin as a viable treatment for migraine. Intranasal administration has been chosen as the treatment route of choice as oxytocin is a small polypeptide with a short half-life of 3 to 4 min [100], making oral or parenteral administration unfeasible.

##### Preclinical Studies

A preclinical study conducted by Tzabazis et al. [85] has shown intranasal oxytocin to be effective in reaching the trigeminal system and brain regions implicated in migraine and other pain. High levels were detected in all 3 branches of the trigeminal nerve, the trigeminal ganglion, trigeminal nucleus caudalis, as well as other brain areas, including cortical areas, caudate/putamen, septal nucleus, hippocampus, thalamus, hypothalamus and midbrain, and the pons and medulla [85]. Once the route of administration was validated, electrophysiological and gene expression experiments went on to show that intranasal oxytocin is able to attenuate nociceptive response in the trigeminal nucleus caudalis in response to peripheral noxious stimulation. However, it was later determined that efficacy of oxytocin in modulating trigeminal pain is affected by inflammatory states, which have an effect on oxytocin receptor expression in the trigeminal system [85]. For example, in preclinical models of trigeminal nociception where there is pre-existing cranial inflammation, oxytocin exhibited a strong efficacy in attenuating pain behavior *versus* models without inflammation [98], indicating that inflammation is critical in determining the level of analgesic efficacy. This finding may explain the lack of acute efficacy in low-frequency migraineurs and suggests that oxytocin treatment may therefore may be more beneficial in more chronic migraine cases.

##### Clinical Studies

Clinical studies, both case studies and clinical trials, provide good evidence for the further investigation of the therapeutic effect of oxytocin in migraine treatment. In 2006, Phillips et al. [92] reported the beneficial effect of intravenous oxytocin in ameliorating migraine headaches in 2 cases, 1 adult and 1 pediatric. Pain relief in both cases was rapid and temporally related to oxytocin administration.

A clinical trial investigating the effectiveness of oxytocin in migraine was reported in 2017. Tzabazis et al. [101] conducted a pilot double-blind, placebo-controlled, single-dose study to assess the pain relief after giving either placebo (38 subjects) or intranasal oxytocin (42 subjects; Syntocinon NasalVR, 32 IU) at the onset of headache in low-frequency episodic migraineurs. The results showed that there was no statistically significant difference in the pain reduction at 2 h between the oxytocin-treated and control groups (33% and 26%, respectively). Reduction of photophobia and phonophobia was evident in the oxytocin-treated group, but the difference was not statistically significant. However, despite the lack of a statistically significant difference at the 2-h period, there was a strong trend of superiority for the oxytocin-treated active group in terms of subject satisfaction at 24 h.

The same group also conducted the study in patients with chronic migraine. Forty patients with chronic migraine were randomized to receive either 32 IU of intranasal oxytocin (Syntocinon NasalVR; 22 subjects) or a matched placebo intranasal spray (18 subjects). They found that although there was not a significant difference in the proportion of subjects experiencing substantial pain relief (reduction from moderate or severe pain to mild or none) at 2 h), there was a significant difference by 4 h after treatment. Interestingly, the effectiveness of oxytocin was compromised by prior taking of nonsteroidal anti-inflammatory drugs. These series of studies also tested the effect of intranasal oxytocin on migraine frequency. Two hundred and eighteen migraine sufferers (161 high-frequency episodic; 56 chronic migraineurs) were included in a multinational double-blind, placebo-controlled study. One hundred and forty-three subjects were in the oxytocin treatment group and 75 in the placebo treatment group. The intervention consisted of a 28-day baseline phase followed by 56 days of “as needed” dosing with either 30 IU of intranasal oxytocin or matching placebo. The primary endpoint was the reduction in migraine headache days from the baseline period to the final 28 days. The results showed a clear reduction in headache frequency as well as responder rate in the oxytocin treatment group. Unfortunately, the study did not meet the primary endpoint owing to an extremely high placebo rate at one study site. The authors concluded that their studies provide a strong argument for further development of intranasal oxytocin for migraine prophylaxis.

### PACAP

#### PACAP Physiology

PACAP is a neuropeptide that was first identified from the hypothalamus of sheep [102]. PACAP belongs to the same family as vasoactive intestinal polypeptide (VIP), glucagon, and secretin. PACAP can exist as 2 separate forms, PACAP-38 (a 38-amino acid peptide) and PACAP-27 (a truncated 27-amino acid peptide). Both share 68% homology with VIP at the N-terminal domain [102]. PACAP-38 is more prevalent than PACAP-27 in mammalian tissue [103, 104], and is widely expressed in both the central and peripheral nervous systems. The main functions include neuroprotection, neurotrophism, neurotransmission, neuromodulation, and vasodilation [105–108]. PACAP is distributed at several levels in the ascending and descending pain transmission pathways, suggesting its role in nociception and pain modulation. Immunohistochemical studies revealed a high density of PACAP-immunoreactive fibers in the superficial lamina I and II of the spinal dorsal horn, an area important in nociceptive transmission and modulation of somatosensory information processing [109–112] .

PACAP binds with 3 different receptors, namely pituitary adenylate cyclase activating polypeptide 1 (PAC1), vasoactive intestinal polypeptide receptor 1 (VPAC1), and vasoactive intestinal polypeptide receptor 2 (VPAC2) [113, 114]. Similar to VIP, receptors for PACAP belong to the GPCR family. PACAP binds potently and specifically to the PAC1 receptor, which is coupled to multiple intracellular signaling cascades, including ERK activation and phospholipase C activation [115, 116]. Both PACAP and VIP bind with near-equal affinity to VPAC1 and VPAC2 receptors that are coupled principally to adenylyl cyclase [102, 106, 117]. An *in vitro* study showed that PACAP-responsive receptors in rat trigeminal neurons and glia were pharmacologically distinct. PACAP-38, but not PACAP-27, activated ERK in glia, whereas both forms stimulated cellular cyclic adenosine monophosphate production [116]. All types of PACAP receptors have been found in parasympathetic, sympathetic ganglia, and sensory ganglia in humans [118].

#### PACAP and Migraine

Owing to its vasodilating property and its presence in parasympathetic and specifically trigeminal ganglia [119], PACAP is hypothesized to be involved in the pathogenesis of vascular headache, including migraine. In support of this, an increased level of PACAP, measured in the external jugular vein, was reported during the migraine attacks [120] and compared with headache-free subjects, migraineurs have a lower level of PACAP during the attack-free period, with this level rising substantially during the attack [121]. Furthermore, a negative correlation between PACAP level and attack duration has also been reported [122]. However, 1 study showed no significant change of serum PACAP level in a large series of chronic migraine sufferers [123].

Several studies have shown that administration of PACAP can induce migraine-like headache [124, 125]. Although PACAP-38 infusion caused acute headache and vasodilatation in both healthy subjects and patients with migraine, the delayed migraine-like headache occurred much more frequently in migraine sufferers [124]. PACAP-38-induced cranial vasodilation was long lasting (> 2 h) and confined solely to extracranial arteries [125]. This incidence of migraine-like headache tends to relate with the dose of PACAP-38 [126]. Functional magnetic resonance imaging showed that PACAP-38-induced migraine attacks are associated with alteration in brain connectivity. An increase in connectivity was seen in the bilateral opercular part of the inferior frontal gyrus, the right premotor cortex, left primary auditory, secondary somatosensory, premotor, and visual cortices. Decreased connectivity was observed in the left visual cortex, right cerebellum, and left frontal lobe [127]. A recent study in patients with migraine without aura showed that PACAP-38 infusion elevated the plasma levels of VIP, prolactin, S100 calcium binding protein, and thyroid-stimulating hormone but not CGRP and tumor necrosis factor-α. There was no association between the development of delayed migraine-like attacks or the presence of the *MEF2D* gene variant (the mutation that increases risk of migraine without aura) with preictal changes in plasma levels of neuropeptides, tumor necrosis factor-α, and pituitary hormones [128, 129].

#### Migraine Drugs Targeting PACAP

Considering that PACAP-38 is a trigger of migraine attacks, antagonizing PACAP receptors, especially PAC1 receptor, can be a potential mechanism for antimigraine drugs [130, 131].

##### Preclinical Studies

Preclinical studies confirm the role of PACAP in controlling cranial vascular tone. Electrical stimulation of nerves in the superior sagittal sinus increased levels of PACAP and CGRP in the cranial circulation in the cat [120]. PACAP-38 administered in increasing concentrations caused a concentration-dependent CGRP-release in the trigeminal nucleus caudalis but not in trigeminal ganglion (TG). The PACAP-38 induced CGRP release is not mediated via the PAC1 receptor as it cannot be altered by PAC1 receptor agonist maxadilan or the PAC1 antagonist M65 [132].

Both VIP and PACAP-38 cause short-lived meningeal vasodilation mediated by VPAC2 receptors, which did not coincide with activation of central trigeminovascular neurons. Administration of PACAP_6–38_, a PAC1 receptor antagonist, significantly inhibited neurogenic dural vasodilation. Given that the PAC1 receptor is not responsible for direct vasodilatory actions on the vessels, this inhibition is likely to be mediated via PAC1 receptors located on presynaptic nerve terminals of the trigeminal innervation of the dural vasculature [133]. The PAC1 receptor may also be responsible for PACAP-38 induced dilatation of the middle meningeal artery [134].

On the neuronal side, PACAP-38 causes delayed activation and sensitization of central trigeminovascular neurons. After a 90-min delay, PACAP-38 increased ongoing spontaneous firing and hypersensitivity to intra- and extracranial somatosensory stimulation without a late response of meningeal artery vasodilation. The dural nociceptive-evoked action potentials in central trigeminovascular neurons was inhibited only by intracerebroventricular administration of the PAC1 receptor antagonist [133]. Microinjection of PACAP-38 into the paraventricular nucleus of the hypothalamus enhanced spinal trigeminal sensory nucleus caudalis basal activity, and this enhancing effect was blocked by a PACAP_6–38_ receptor antagonist [15].

##### Clinical Studies

Evidence confirming clinical efficacy of drug affecting PACAP in migraine treatment is not present at this moment. A phase IIa randomized, double-blind, placebo-controlled study to evaluate the efficacy and safety of AMG 301, a PAC1 receptor monoclonal antibody, in migraine prevention has been registered but has not yet recruited (ClinicalTrials.gov Identifier: NCT03238781).

### NPY

#### NPY Physiology

NPY is a 36-amino acid peptide hormone that is expressed in the central and peripheral nervous systems [135, 136]. In the central nervous system, NPY is expressed in the cell bodies of neurons and is most highly concentrated in the cerebral cortex, brainstem, and hypothalamic nuclei (paraventricular and ventromedial nuclei, and the lateral hypothalamus) [137–139]. NPY is considered to play an important role in multiple physiological processes, including food intake, cognition, epileptic seizure activity, learning, stress sensitivity, and mood [140, 141]. In the peripheral nervous system, NPY is expressed in sympathetic postganglionic neurons, chromaffin cells or pheochromocytes of the adrenal medulla, platelets, and adipose tissue [142–144] . NPY is co-localized with tyrosine hydroxylase, suggesting its involvement in cardiovascular control [145, 146].

NPY receptors are a group of GPCRs, which are classified into 5 subtypes known as Y1, Y2, Y4, Y5, and Y6 [147]. The Y1 and Y2 receptors have been the most intensively investigated receptors in studies of nociception [148, 149]. NPY receptors are present in the central nervous system, including in the trigeminal ganglion and caudal trigeminal nucleus, suggesting a role in migraine pathophysiology. NPY receptors are present on distinct populations of sensory neurons, and receptor activation can modulate the activity of nociceptive neurons. Activated NPY receptor inhibits adenylate cyclase through inhibitory G proteins leading to reduced cyclic adenosine monophosphate levels in target cells [150, 151].

#### NPY and Migraine

The premonitory symptoms in migraine such as changes in appetite have been proposed to involve NPY [5, 152] and NPY has been demonstrated in human cranial vessels, reflecting its role in controlling cranial vasculature and its implication in migraine pathogenesis [153, 154]. Despite this, studies investigating the association between level of NPY and migraine showed conflicting results. A study in juvenile migraine (both with and without aura) showed significantly lower plasma levels of NPY in the interictal period, with respect to the control group. Plasma NPY levels tended to significantly increase during attacks in patients with migraine with aura [155]. On the contrary, Goadsby et al. [156] showed that the NPY immunoreactivity in the external jugular venous blood did not alter during migraine attacks in patients with migraine with or without aura [156]. Vecsei et al. [157] also reported that the NPY concentrations in plasma of the patients with migraine during the attack and attack-free period did not differ significantly from each other, or from the “mixed neuropsychiatric group” [157].

The data concerning the alteration of level of NPY in the CSF of migraineurs are also inconclusive. The NPY immunoreactivity in the CSF was reported to be higher in migraineurs during the attacks than in controls [158], whereas another research group did not observe an NPY immunoreactivity elevation in the suboccipital CSF plasma during attacks and attack-free periods of patients with migraine without aura [157].

NPY may be involved in pathogenesis of weight gain seen in patients with migraine under preventive medication. Plasma NPY levels in migraine patients taking flunarizine or amitriptyline were markedly increased, with the highest levels during the second and third months [159]. The mechanism underlying this drug-induced weight gain may involve the alteration of leptin transport system or leptin sensitivity.

#### Migraine Drugs Targeting Hypothalamic NPY

As of yet, no drugs have been developed specifically or otherwise applied to targeting the NPY system in migraine; however, preclinical studies antagonizing the Y1 receptor show that this may prove to be a potential therapeutic target.

##### Preclinical Studies

NPY is localized in the superficial laminae of the spinal dorsal horn and inhibits nociceptive processing at this site. In the dorsal root ganglia in rats, Y1 receptors are extensively co-localized with CGRP or substance P (SP). Y1 receptors contribute to the antihyperalgesic effects of NPY by inhibiting SP release, and Y1 receptor signaling in the dorsal horn is increased during inflammatory nociception [160]. In rats, activation of the Y1 receptor can inhibit capsaicin-sensitive nociceptors in the spinal cord or hindpaw tissue [161, 162].

Y1 receptors are enhanced after intraplantar injection of complete Freund’s adjuvant in rat. NPY administered intrathecally in rats inhibits hyperalgesia associated with nerve injury and inflammation, and the expression of Fos, a protein marker of neuronal activity [163–166]. Both of these effects of NPY were blocked by Y1 receptor antagonists suggesting NPY plays a role in nociceptive transmission.

NPY acts on Y1 receptors in the spinal dorsal horn to decrease nociception by inhibiting SP release, and that this effect is increased by inflammation [148, 167]. NPY decreases capsaicin-evoked SP-like immunoreactivity in microdialysate from the dorsal horn. Systemic administration of NPY and a Y1 receptor agonist inhibited dural stimulus-evoked and spontaneous neuronal firing in the trigeminocervical complex. However, Y2 and Y5 receptor agonists, and a Y1 receptor antagonist had no significant effects on dural stimulus-evoked or spontaneous neuronal firing in the trigeminocervical complex [168].

##### Clinical Studies

Unfortunately, no clinical trial investigating effectiveness of NPY-related compounds in migraine has been conducted.

## Concluding Remarks

**Fig. 1:**
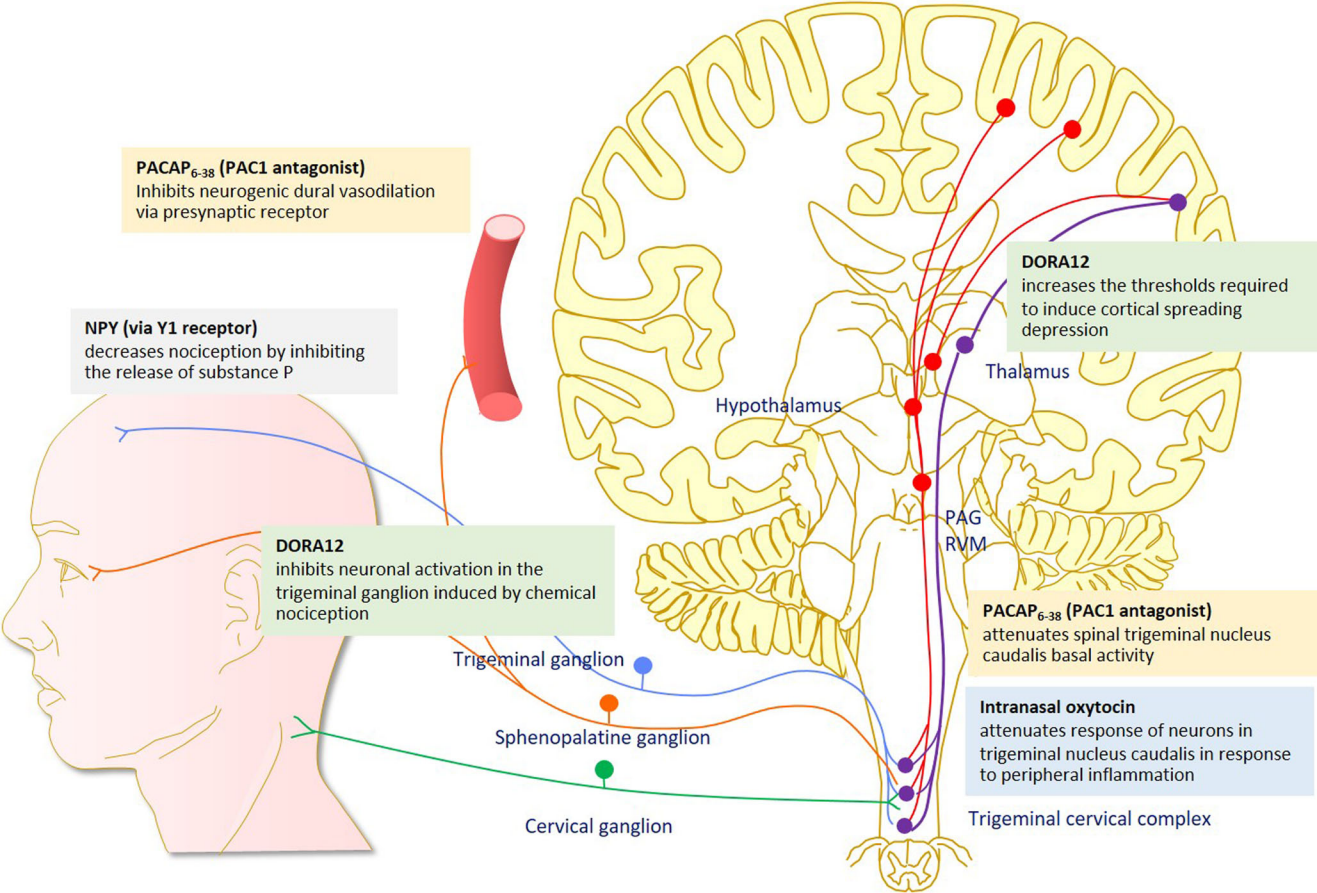
The effect of hypothalamic pharmacological interventions on migraine-related brain processing. PACAP = pituitary adenylate cyclase activating protein; PAC1 = pituitary adenylate cyclase-activating polypeptide 1; DORA = dual orexin receptor antagonist; PAG = periaqueductal grey; RVM = rostral ventromedial medulla

The prominent role that the hypothalamus and its peptide systems have in migraine pathophysiology is becoming increasingly clear. The above evidence, mainly preclinical, suggests that modification of hypothalamic peptides may be potential targets in migraine pharmacotherapy (Fig. 1, Table 1). At this moment, information regarding clinical efficacy is still very limited. Although targeting hypothalamic peptides in migraine drug development is theoretically sound, the issue of adverse effects should be considered. Hypothalamic peptides are usually involved in a wide range of homeostatic control; therefore, using drugs targeting these peptides may have some limitation or undesirable adverse effects. For example, in the case of orexin, the sedative effect of filorexant limits the use to be only once daily (before bedtime) to avoid daytime somnolence. This drug administration may explain the negative results of the study [80], and further investigation into orexin therapies should not be discounted. Oxytocin and PACAP show promising therapeutic efficacy and the results of the PACAP clinical trial are much anticipated. Additionally, other targets, such as NPY have shown inconclusive preclinical results regarding attenuation of trigeminal nociception; however, there remains a strong clinical association and most notably in the premonitory stage, suggesting a possible therapeutic application when applied to other phases of the migraine attack.

**Table 1:**
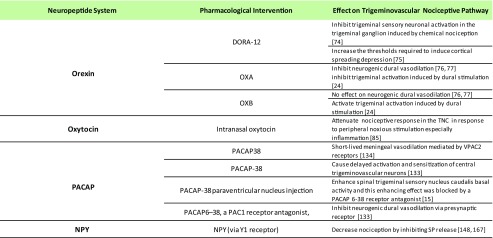
Preclinical evidence for hypothalamic neuropeptides effect on the trigeminovascular nociceptive pathway

In addition to the above, other related peptides such as melatonin and melanin-concentrating hormone (MCH) may also play a role in controlling the excitability of trigeminal nociceptive neurons and thereby may be involve in the migraine pathogenesis. Melatonin is synthesized in the pineal gland rather than hypothalamus, but is involved in the circadian regulation of sleep [169] and has been postulated to play a role in migraine. Melatonin levels are seen to be decreased in patients with migraine [170], and a recent clinical study showed that melatonin 3 mg is better than placebo for migraine prevention, and more tolerable and effective than the migraine preventative amitriptyline (25 mg) [171]. MHC, however, is another hypothalamic hormone that has been proposed to involve in the interactions between food intake, drowsiness, and migraine. This hypothesis is based on the findings of the presence of hypothalamic MCH terminals on thalamic trigeminovascular neurons; however, this system has not yet been targeted pharmacologically with regard to migraine or other headaches [172], but further investigation may reveal a role for MCH in migraine pathophysiology.

In conclusion, it is clear that perturbed hypothalamic peptide networks play a role in migraine pathogenesis from the earliest stage of the attack. Evidence that these peptides are able to modulate trigeminovascular nociception and thus the potential remains to develop new therapies to alleviate head pain. However, where the true potential lies is in the ability to identify and target the hypothalamic-driven premonitory phase, by focusing our attention on better understanding these early mechanisms there is great opportunity to stop migraine in its tracks.

### Required Author Forms

Disclosure forms provided by the authors are available with the online version of this article.

## Electronic supplementary material

ESM 1 (PDF 1224 kb)
